# Prognostic value of postoperative dynamic PNI trajectories for biochemical recurrence in patients with high-risk localized prostate cancer: a longitudinal trajectory clustering study

**DOI:** 10.3389/fnut.2026.1963784

**Published:** 2026-10-06

**Authors:** Bo Yu, Xuchao Dai, Jianguo Zhu

**Affiliations:** 1 Department of Urology, The Second Affiliated Hospital of Zunyi Medical University, Zunyi, China; 2 Department of Urology, Guizhou Medical University Affiliated Hospital, Guiyang, China; 3 Department of Urology, Guizhou Provincial People's Hospital, Guiyang, China; 4 Graduate School, Zunyi Medical University, Zunyi, China

**Keywords:** biochemical recurrence, high-risk localized prostate cancer, longitudinal trajectory clustering, prediction model, prognostic nutritional index

## Abstract

**Background:**

The prognostic nutritional index (PNI) reflects nutritional-immune status, but the value of a single baseline PNI for predicting biochemical recurrence (BCR) after radical prostatectomy in high-risk localized prostate cancer remains unclear. We aimed to identify postoperative PNI trajectories and compare their BCR predictive value with baseline PNI.

**Methods:**

We retrospectively included 436 patients. PNI was measured preoperatively (T0) and at 3 (T1) and 12 (T2) months post-surgery. Longitudinal trajectory clustering identified three PNI trajectory classes: persistently high (PHigh, *n* = 169, 46.4%), persistently low (PLow, *n* = 146, 40.1%), and declining (Declining, *n* = 49, 13.5%). Cox regression assessed associations with BCR; a Cox-based time-to-event prediction model was evaluated using time-dependent AUC, IPCW Brier score, and calibration at 3-year and 5-year horizons. A 12-month landmark analysis was performed to address potential guarantee-time bias.

**Results:**

During median 38.2-month follow-up, 122 (28.0%) developed BCR. Baseline PNI was not independently associated with BCR (continuous HR = 0.97, *p* = 0.320; dichotomized HR = 1.23, *p* = 0.347). Three trajectories emerged: persistently high (PHigh, 46.4%), persistently low (PLow, 40.1%), and declining (Declining, 13.5%). Relative to PHigh, PLow (HR = 2.95) and Declining (HR = 5.80) both had significantly higher BCR risk (*p* < 0.001). A 12-month landmark analysis confirmed these findings (PLow HR = 2.72, *p* = 0.002; Declining HR = 4.79, *p* = 1.24 × 10^−6^). The multivariable Cox model combining trajectory, log PSA, surgical margin, and nodal metastasis achieved a 3-year time-dependent AUC of 0.823 (optimism-corrected 0.815) and a 5-year AUC of 0.790 (optimism-corrected 0.781). The Brier score was 0.117 at 3 years (25.8% improvement over null) and 0.166 at 5 years; the calibration slope was 1.057 (optimism-corrected 0.989) at 3 years and 1.001 (optimism-corrected 0.940) at 5 years.

**Conclusion:**

Postoperative PNI follows three distinct change-pattern subgroups. Dynamic PNI trajectories are strongly associated with BCR risk, whereas a single baseline PNI is not. Patients with persistently low or declining PNI are at high risk, supporting routine PNI monitoring to guide individualized follow-up. These observational findings require prospective multicenter validation.

## Introduction

Prostate cancer is the second most common malignancy among men worldwide. In China, the incidence of prostate cancer has been rising steadily due to the widespread use of PSA screening and population aging (1, 2). Although high-risk localized prostate cancer remains confined to the organ, it poses a major clinical challenge because of its high aggressiveness and recurrence risk (3, 4). Radical prostatectomy is an important treatment for these patients, but about 20–40% of patients experience biochemical recurrence (BCR) after surgery, defined as a persistent rise in PSA levels, which serves as an early warning sign of local recurrence or distant metastasis (5, 6). BCR not only seriously impairs quality of life but also correlates closely with long-term cancer-specific mortality (7). Therefore, accurate identification of those at high risk of BCR is essential for designing individualized postoperative follow-up and adjuvant therapy strategies (8). Currently, clinical risk stratification relies mainly on tumor-related factors such as PSA level, pathological T stage, ISUP grade, and surgical margin status. However, these indicators largely reflect the intrinsic biological features of the tumor and do not fully incorporate information on the host systemic nutritional-inflammatory status that modulates tumor prognosis (9, 10).

The preoperative prognostic nutritional index (PNI) is a composite indicator calculated from serum albumin and peripheral blood lymphocyte count, using the formula: serum albumin (g/L) + 5 × peripheral blood lymphocyte count (×10^9^/L) (11). This index captures both the nutritional reserve and immune competence of the host. It has been validated as a reliable prognostic biomarker in various solid tumors, including gastric, colorectal, and lung cancers (12). In prostate cancer, several studies have shown that a low preoperative PNI is associated with an increased risk of postoperative BCR and worse overall survival (13). However, almost all existing studies have relied on a single baseline (preoperative or immediate postoperative) PNI measurement, treating PNI as a static, time-fixed characteristic (14). This assumption overlooks the dynamic evolution of nutritional and immune status during the perioperative and postoperative recovery phases. Factors such as surgical trauma stress, anesthetic effects, metabolic disturbances during recovery, implementation of adjuvant therapy, and changes in tumor burden can all significantly influence serum albumin levels and lymphocyte counts. A single baseline measurement is like a snapshot; it can reflect the instantaneous state before surgery but cannot capture the fluctuating trajectory or long-term trend of postoperative PNI. This may be the fundamental reason why the effect of baseline PNI is often unstable after multivariable adjustment (15). In fact, tumor progression is a dynamic process of continuous interaction between cancer cells and the host microenvironment, and static indicators may seriously underestimate the true prognostic contribution of host factors.

In recent years, longitudinal trajectory clustering has emerged as a powerful methodological tool for analyzing longitudinal biomarkers. It can identify heterogeneous subgroups with similar change patterns based on repeated measurements from individuals, thus revealing latent risk categories that traditional time-dependent covariate models cannot detect (16, 17). Based on this, we hypothesized that postoperative PNI in patients with high-risk localized prostate cancer is not homogeneous over time but exhibits distinct dynamic trajectory subtypes, and that the longitudinal trend of PNI has stronger predictive value for BCR than a single baseline measurement. To test this hypothesis, we retrospectively enrolled 436 patients with high-risk localized prostate cancer, collected PNI data at three time points (immediate postoperative T0, 3 months T1, and 12 months T2), used longitudinal trajectory clustering to identify PNI trajectory classes, systematically compared the predictive performance of baseline PNI versus PNI trajectories for BCR, and constructed a nomogram prediction model incorporating dynamic trajectories. This study aimed to break the static biomarker paradigm, provide a novel dynamic assessment strategy for postoperative risk stratification in prostate cancer, and offer evidence-based support for the timing of individualized nutritional-immune interventions.

## Materials and methods

### Study design and population

This was a single-center retrospective cohort study, approved by the Ethics Committee of the Affiliated Hospital of Guizhou Medical University (approval no. 2026-06-55). We enrolled 436 patients with high-risk localized prostate cancer who underwent radical prostatectomy. Exclusion criteria included: distant metastasis (M1 stage), receipt of palliative treatment, perioperative severe complications leading to loss of follow-up, or missing key variables. The primary endpoint was biochemical recurrence (BCR), defined as two consecutive postoperative PSA levels ≥0.2 ng/mL with an upward trend. BCR time was calculated from the date of surgery to the date of first PSA elevation (18). During follow-up, 122 BCR events (28.0%) were recorded. The median follow-up time was 38.2 months (range, 1–90 months).

### Data collection and PNI assessment

The prognostic nutritional index (PNI) was calculated as: PNI = serum albumin (g/L) + 5 × peripheral blood lymphocyte count (×10^9^/L) (11). Preoperative PNI (PNI_base) was measured from fasting venous blood samples collected within 1 week before surgery. Postoperative PNI values were obtained at follow-up visits at 3 months (T1) and 12 months (T2) after surgery, together with the preoperative baseline (T0), forming three longitudinal measurement time points. The mean PNI_base in the whole cohort was 49.4 (median 49.4; range 39.8–60.0).

Baseline data covered the following domains: demographic characteristics (age, BMI); tumor characteristics (preoperative PSA, clinical T stage, pathological T stage, ISUP grade, surgical margin, seminal vesicle invasion, lymph node metastasis, lymphovascular invasion); treatment information (neoadjuvant ADT, adjuvant therapy); and laboratory parameters (serum albumin, peripheral blood lymphocyte count). According to the optimal cut-off value of 46.5 determined by X-tile software, patients were divided into a low PNI group (PNI < 46.5, *n* = 94) and a high PNI group (PNI ≥ 46.5, *n* = 342) (19).

### Association of baseline PNI with BCR

We evaluated the relationship between baseline PNI and BCR from four aspects. (1) Continuous variable analysis: PNI_base was entered as a continuous independent variable into univariable and multivariable Cox proportional hazards regression. The multivariable model adjusted for age, log PSA, pathological T stage, ISUP grade, surgical margin, seminal vesicle invasion (SVI), lymph node metastasis (LNI), and lymphovascular invasion (LVI). Covariate selection was based on the results of univariable Cox regression and consensus prognostic factors for BCR after radical prostatectomy in high-risk localized prostate cancer, and strictly followed the rule of at least 10 events per variable (EPV) to avoid overfitting (20). Hazard ratios (HR) and 95% confidence intervals (CI) were reported. (2) Dichotomized analysis: using the threshold of 46.5, we divided patients into low and high PNI groups, estimated BCR-free survival curves using the Kaplan–Meier method, and compared groups with the log-rank test. (3) Nonlinear dose–response analysis: restricted cubic splines (RCS) with 4 knots at the 5th, 35th, 65th, and 95th percentiles of PNI were used to test the nonlinear relationship between PNI_base and BCR risk, with the median (49.4) as the reference (21). (4) Proportional hazards assumption: we tested the proportional hazards assumption for each covariate and the overall model using Schoenfeld residuals (22).

### Subgroup analyses

Using PNI_base as a continuous exposure variable, we evaluated the consistency of the prognostic effect of baseline PNI across 12 predefined clinical subgroups: age (<65 vs. ≥ 65 years), BMI (<24 vs. ≥ 24), PSA (<10 vs. ≥ 10 ng/mL), clinical T stage (T1-T2 vs. T3), pathological T stage (T2 vs. T3), ISUP grade (GG1-3 vs. GG4-5), surgical margin (negative vs. positive), seminal vesicle invasion (no vs. yes), lymph node metastasis (no vs. yes), lymphovascular invasion (no vs. yes), neoadjuvant ADT (no vs. yes), and adjuvant therapy (no vs. yes). Within each subgroup, we performed multivariable Cox regression to estimate the adjusted HR and 95% CI for PNI_base, adjusting for the same 8 clinical prognostic factors (age, log PSA, pathological T stage, ISUP grade, surgical margin, SVI, LNI, LVI). When the subgroup stratifying variable itself was one of these 8 adjustment variables (e.g., age, PSA, pathological T stage, ISUP grade, surgical margin, SVI, LNI, LVI), we removed that variable from the adjustment set for that subgroup to avoid collinearity and overadjustment. Interaction between subgroups was tested using the likelihood ratio test, comparing the full model (PNI_base × subgroup + adjustment variables) with the reduced model (PNI_base + subgroup + adjustment variables).

### Modeling of dynamic PNI trajectories

For the 364 patients with complete PNI measurements at all three time points (T0, T1, and T2), we performed longitudinal trajectory clustering to identify PNI patterns over time (16, 17). As each patient had only three time points, individual trajectories were fitted using a linear model (PNI ~ time), and we extracted the intercept and slope as trajectory features for each patient. These standardized features were then subjected to Gaussian mixture model (GMM) clustering, with models fitted for 1–6 classes. Model fit was evaluated using the Bayesian Information Criterion (BIC), Akaike Information Criterion (AIC), and sample-size-adjusted BIC (aBIC). BIC was calculated as BIC = −2 × log-likelihood + *k* × ln(*n*), where *k* denotes the number of free parameters and *n* the sample size; lower BIC values indicated better fit. The bootstrap likelihood ratio test (BLRT, with 200 bootstrap samples) served as the primary statistical test for comparing a k-class model against a (*k*−1)-class model. Classification quality was assessed by entropy (>0.8 acceptable), average posterior probability (AvePP, >0.7 acceptable), and the odds of correct classification (OCC, >5 acceptable). A minimum class proportion exceeding 5% was also considered acceptable. The final model was chosen through a comprehensive evaluation of statistical fit, classification quality, and sample size adequacy to ensure reliable prognostic estimation.

### Association of trajectory classes with BCR

We estimated BCR-free survival probabilities for the three trajectory groups using the Kaplan–Meier method and plotted survival curves; group differences were assessed by the log-rank test, with pairwise comparisons adjusted using Bonferroni correction. Cox proportional hazards regression was used to estimate the HR and 95% CI for BCR in the PLow and Declining groups relative to the PHigh group, with both unadjusted and adjusted models (adjusting for age, log PSA, pathological T stage, ISUP grade, surgical margin, SVI, LNI, LVI). The proportional hazards assumption was checked using Schoenfeld residuals. Baseline characteristics were also described by trajectory subgroups to evaluate clinicopathological differences among groups.

### Prediction performance evaluation

We constructed a Cox proportional hazards prediction model with the primary outcome of BCR-free survival, evaluated at clinically meaningful time horizons. Given the median follow-up of 38.2 months, we selected 3-year BCR as the primary prediction horizon and 5-year BCR as a secondary horizon. The four predictor variables (PNI trajectory class, log PSA, surgical margin, and lymph node metastasis) were pre-specified based on established prognostic consensus for BCR after radical prostatectomy and the events-per-variable (EPV ≥ 10) principle, rather than *post-hoc p*-value significance. Discrimination was assessed using time-dependent AUC (cumulative/dynamic) via the timeROC R package, which properly handles censored observations through inverse probability of censoring weighting (IPCW). Bootstrap 95% confidence intervals (*B* = 500) were reported.

Based on the optimal model, we constructed a Cox-based nomogram for individualized prediction of 3-year BCR-free probability (23, 24). The IPCW Brier score was computed over time using the pec package. Calibration at 3 years and 5 years was assessed using the rms::calibrate function with the Kaplan–Meier method (*m* = 30 per group, *B* = 1,000); calibration slope and intercept were reported. Bootstrap optimism correction (*B* = 500) was applied to the 3-year time-dependent AUC: the model was refit on each bootstrap sample, and the difference between the bootstrap-sample AUC and the original-sample AUC (using the bootstrap model) was averaged to estimate optimism. The optimism-corrected AUC was computed as apparent AUC minus mean optimism. Decision curve analysis (DCA) was used to evaluate the net clinical benefit of the model across a threshold probability range of 5–50%.

### Sensitivity analyses

To test the robustness of our findings, we conducted the following sensitivity analyses: (1) re-evaluating the effects of baseline PNI and trajectory classes after excluding patients who received neoadjuvant ADT; (2) re-analyzing after excluding patients who received adjuvant therapy; (3) performing a complete-case analysis by including only the 364 patients with complete trajectory data, re-estimating the effects of baseline PNI and trajectory classification; and (4) a 12-month landmark analysis to address potential guarantee-time bias. In this analysis, survival time was redefined from the 12-month landmark, excluding patients who experienced BCR (*n* = 18) or were censored (*n* = 6) before 12 months.

### Statistical analysis

Continuous variables were presented as medians with interquartile ranges, and group comparisons were performed using the Mann–Whitney *U* test (two groups) or Kruskal–Wallis test (three or more groups). Categorical variables were presented as counts (percentages) and compared using the chi-square test or Fisher’s exact test. Baseline characteristics tables were generated using the tableone package, stratified separately by PNI dichotomous groups (low vs. high) and by trajectory subgroups (PHigh/PLow/Declining). All analyses were performed in R version 4.3.1 (R Foundation for Statistical Computing). A two-sided *p* < 0.05 was considered statistically significant unless otherwise specified.

## Results

### Baseline characteristics of the study population

A total of 436 patients with high-risk localized prostate cancer were included, and the overall BCR rate was 28.0% (122/436). The median age was 69.0 years (IQR: 65.0–74.0), median BMI was 23.8 (22.1–25.5), and median preoperative PSA was 16.7 ng/mL (10.4–27.3). Regarding pathological features, high ISUP grade (GG4-5) accounted for 45.9%, positive surgical margin rate was 24.5%, seminal vesicle invasion 14.2%, lymph node metastasis 5.7%, and lymphovascular invasion 19.5%. The median follow-up time was 38.2 months (range 1–90 months) (Table 1).

**Table 1:** Baseline characteristics of high-risk localized prostate cancer patients (by PNI group).

Characteristic	Overall (*n* = 436)	Low PNI (*n* = 94)	High PNI (*n* = 342)	*p* value
Age (years)	69.0 (65.0–74.0)	72.0 (67.0–75.8)	69.0 (65.0–73.0)	<0.001
BMI	23.8 (22.1–25.5)	23.9 (21.9–25.5)	23.8 (22.1–25.5)	0.987
Preoperative PSA (ng/mL)	16.7 (10.4–27.3)	20.2 (11.3–30.8)	15.4 (10.2–26.2)	0.026
Serum albumin (g/L)	40.3 (38.3–42.2)	37.2 (35.8–38.4)	41.0 (39.3–42.8)	<0.001
Lymphocyte count (×10^9^/L)	1.8 (1.5–2.1)	1.4 (1.2–1.6)	1.9 (1.7–2.2)	<0.001
PNI_base	49.4 (46.9–52.0)	44.8 (43.4–45.7)	50.4 (48.5–52.6)	<0.001
ISUP grade GG4-5	200 (45.9)	68 (72.3)	132 (38.6)	<0.001
Positive surgical margin	107 (24.5)	18 (19.1)	89 (26.0)	0.216
Seminal vesicle invasion	62 (14.2)	15 (16.0)	47 (13.7)	0.706
Lymph node invasion (LNI)	25 (5.7)	11 (11.7)	14 (4.1)	0.010
Lymphovascular invasion (LVI)	85 (19.5)	21 (22.3)	64 (18.7)	0.523
Neoadjuvant ADT	59 (13.5)	11 (11.7)	48 (14.0)	0.678
Adjuvant therapy	57 (13.1)	13 (13.8)	44 (12.9)	0.942
BCR rate	122 (28.0)	36 (38.3)	86 (25.1)	0.017

Continuous variables are presented as median (interquartile range); categorical variables as *n* (%). PNI, prognostic nutritional index; BCR, biochemical recurrence; ADT, androgen deprivation therapy.

According to the preoperative PNI threshold of 46.5, patients were divided into a high PNI group (*n* = 342) and a low PNI group (*n* = 94); baseline characteristics are shown in Table 1. The low PNI group had older age (median 72.0 vs. 69.0 years, *p* < 0.001), higher preoperative PSA (median 20.2 vs. 15.4 ng/mL, *p* = 0.026), and significantly lower serum albumin (median 37.2 vs. 41.0 g/L, *p* < 0.001) and peripheral blood lymphocyte count (median 1.44 vs. 1.94 × 10^9^/L, *p* < 0.001). Regarding pathological features, the low PNI group had a significantly higher proportion of high ISUP grade (GG4-5) (72.3% vs. 38.6%, *p* < 0.001) and a higher rate of lymph node metastasis (11.7% vs. 4.1%, *p* = 0.010). The BCR rate in the low PNI group was significantly higher than that in the high PNI group (38.3% vs. 25.1%, *p* = 0.017). No significant differences were observed between the two groups in BMI, surgical margin, seminal vesicle invasion, lymphovascular invasion, neoadjuvant ADT, or adjuvant therapy.

### Limited prognostic value of single baseline PNI

We first evaluated the association of a single baseline PNI measurement with BCR. When PNI_base was entered as a continuous variable in univariable Cox regression, the unadjusted HR was 0.94 (95% CI: 0.89–0.98, *p* = 0.008), indicating that each 1-unit increase in baseline PNI was associated with about a 6% reduction in BCR risk. However, after adjustment for age, log PSA, pathological T stage, ISUP grade, surgical margin, SVI, LNI, and LVI, the independent effect of PNI_base was no longer significant (HR = 0.97, 95% CI: 0.92–1.03, *p* = 0.320). Using the threshold of 46.5, the low PNI group showed a significantly increased BCR risk in univariable analysis (HR = 1.66, 95% CI: 1.13–2.46, *p* = 0.010), but the effect was not significant after multivariable adjustment (HR = 1.23, 95% CI: 0.80–1.90, *p* = 0.347).

Kaplan–Meier survival analysis showed that the low PNI group had a significantly lower BCR-free survival rate than the high PNI group (log-rank *p* = 0.010; univariable Cox HR = 1.66, 95% CI: 1.13–2.46) (Figure 1A). Restricted cubic spline analysis (Figure 1B) further examined the nonlinear dose–response relationship between PNI_base and BCR (4 knots), and the results showed no significant dose–response relationship. In summary, a single baseline PNI measurement was not an independent predictor of BCR risk after multivariable adjustment, and its static cut-off value had limited utility in prognostic assessment.

**Figure 1:**
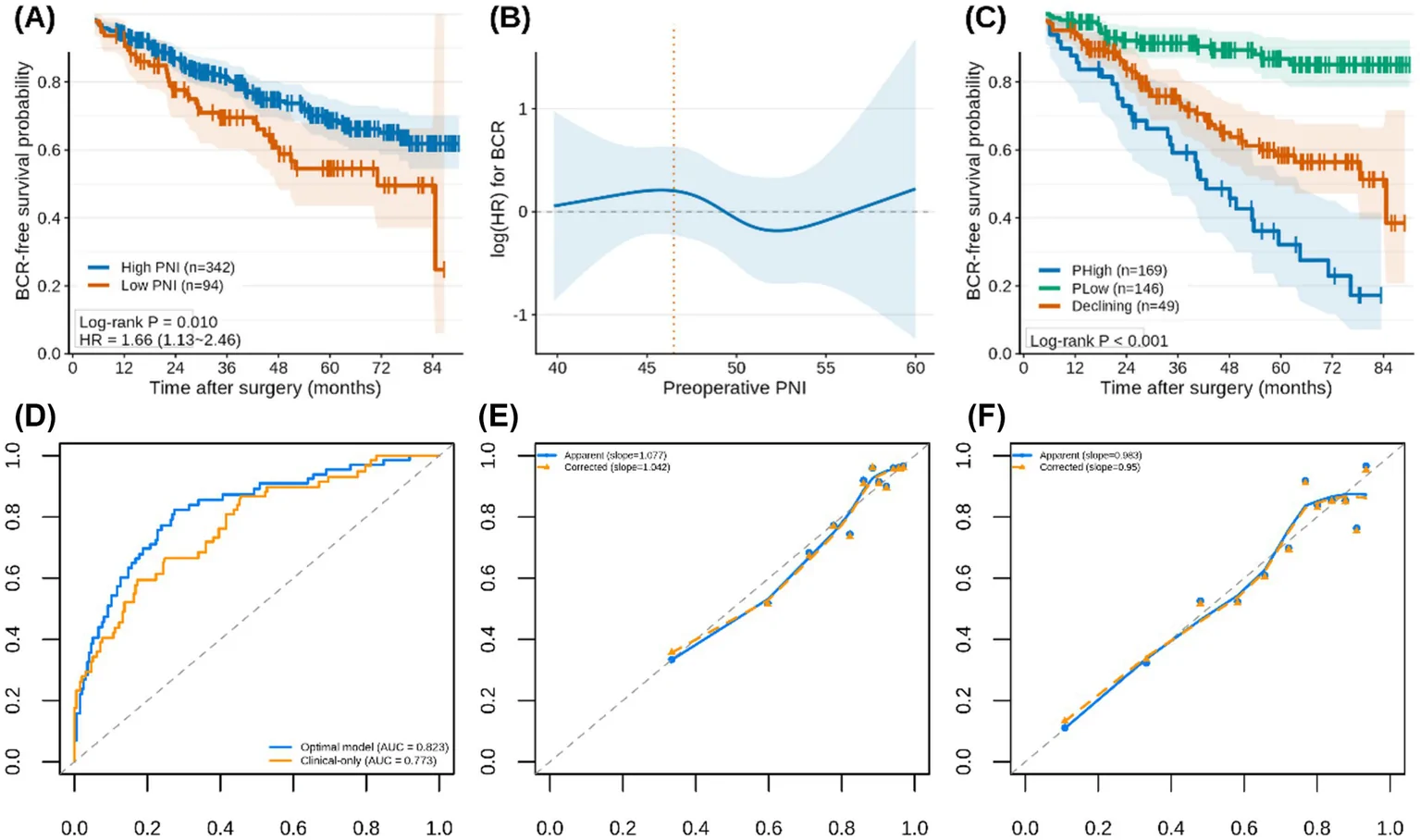
Relationship between baseline PNI, longitudinal PNI trajectories, and BCR. **(A)** Kaplan–Meier BCR-free survival curves for the low-PNI vs. high-PNI groups. **(B)** Restricted cubic spline dose–response curve for baseline PNI and BCR risk, with the median (49.4) as the reference. **(C)** Kaplan–Meier BCR-free survival curves for the three trajectory groups (PHigh, PLow, Declining). **(D)** Time-dependent ROC curves at 3 and 5 years for the optimal model versus the clinical-only model. **(E)** Calibration curves at 3 years: apparent (solid blue) and bootstrap-corrected (dashed orange), with the diagonal representing ideal calibration. **(F)** Calibration curves at 5 years: apparent (solid blue) and bootstrap-corrected (dashed orange), with the diagonal representing ideal calibration.

### Subgroup analyses

Using PNI_base as a continuous exposure variable, we evaluated the consistency of the prognostic effect of baseline PNI across 12 predefined clinical subgroups. Within each subgroup, multivariable Cox regression was used to estimate the adjusted HR for PNI_base, adjusting for the 8 clinical prognostic factors (age, log PSA, pathological T stage, ISUP grade, surgical margin, SVI, LNI, and LVI); when the subgroup stratifying variable itself was among these adjustment variables, we removed it from the adjustment set.

The adjusted negative association between baseline PNI and BCR was directionally consistent (HR < 1) in most subgroups, but none reached statistical significance. The most prominent effect was seen in patients receiving neoadjuvant ADT (HR = 0.84, 95% CI: 0.70–1.01, *p* = 0.057), approaching but not reaching the significance threshold. Patients without lymph node metastasis (HR = 0.95, 95% CI: 0.90–1.01, *p* = 0.110) and those aged <65 years (HR = 0.88, 95% CI: 0.75–1.05, *p* = 0.161) showed similar trends. In the remaining subgroups, the adjusted HRs were all close to 1.0, and the 95% CIs crossed the null. All interaction *p* values were >0.05 (range 0.222–0.900), indicating that the effect of baseline PNI was consistent across subgroups without significant interaction. These results suggest that after full adjustment for clinical prognostic factors, the independent prognostic effect of baseline PNI was not significant in any subgroup, further confirming that a single baseline PNI measurement cannot achieve effective risk stratification even in specific subpopulations. The detailed subgroup results are presented in Supplementary Table S2.

### Identification of dynamic PNI trajectories

Given that the static PNI analysis did not reveal a significant independent prognostic association, we next employed trajectory clustering to identify dynamic change patterns using longitudinal PNI measurements from T0 to T2. Among the 364 patients with complete data at all three time points, we compared 1- to 6-class GMM models. Statistically, the 4-class solution was optimal (lowest BIC = 1117.0; BLRT vs. 3-class: *p* < 0.0001), while the 5- and 6-class models showed degraded classification quality (Supplementary Table S1).

However, we retained the 3-class model for the primary analysis based on clinical and methodological considerations. The additional 4th class merely subdivided the PHigh group into two subgroups with virtually identical BCR rates (10.7% vs. 10.6%), providing no incremental prognostic information (LRT *p* = 0.996), and the smallest class (*n* = 28, ~3 BCR events) was insufficient for reliable estimation. The 3-class model demonstrated excellent classification quality (entropy = 1.000, AvePP>0.999, OCC>>5) and yielded three clinically interpretable trajectories: persistently high (PHigh, *n* = 169, 46.4%), persistently low (PLow, *n* = 146, 40.1%), and declining (Declining, *n* = 49, 13.5%). The PHigh group showed rising PNI from 51.6 at T0 to 58.2 at T2; the PLow group maintained stable levels around 46–47; and the Declining group exhibited a sharp drop from 49.9 at T0 to 35.0 at T2 (Figure 2A).

**Figure 2:**
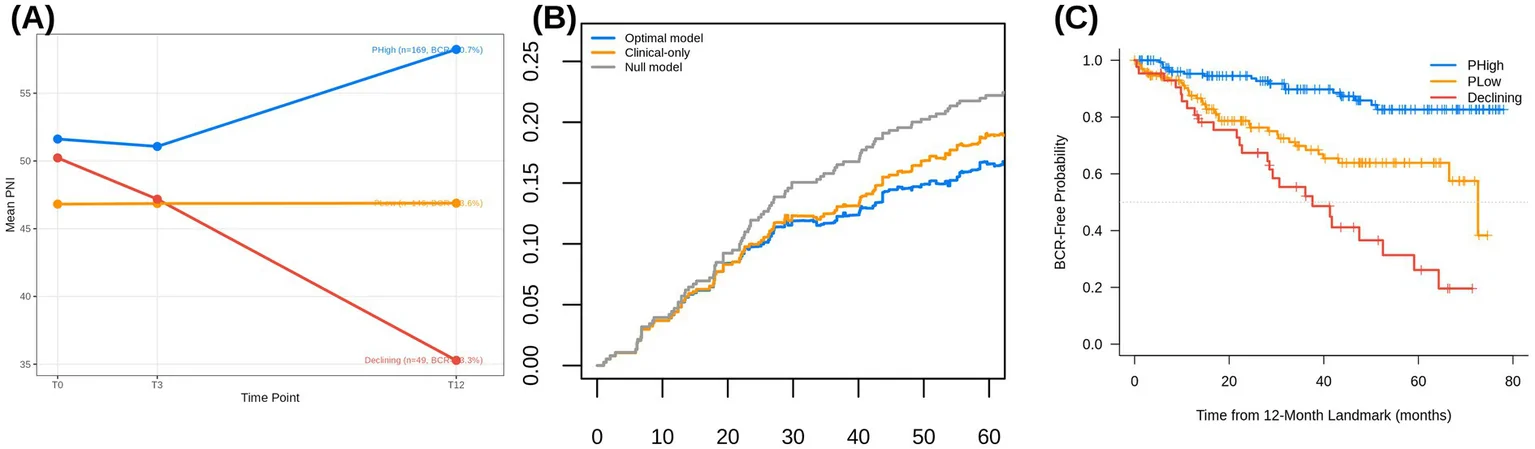
Dynamic PNI trajectory analysis and model validation. **(A)** Three-class PNI trajectory solution showing mean PNI values at T0, T1, and T2 for PHigh, PLow, and Declining groups. **(B)** Brier score over time for the optimal, clinical-only, and null models (IPCW estimator). **(C)** Kaplan–Meier BCR-free survival curves from the 12-month landmark analysis comparing the three trajectory groups.

Baseline characteristics by trajectory subgroup are shown in Table 2. The three groups differed significantly in age (*p* = 0.009) and baseline PNI (*p* < 0.001). The Declining group had a significantly higher rate of positive surgical margins (40.8% vs. 21.9% vs. 22.6%, *p* = 0.019). BCR rates differed strikingly across groups: PHigh 10.7%, PLow 33.6%, and Declining 63.3% (*p* < 0.001). Notably, although the PHigh and Declining groups had similar baseline PNI levels (51.6 vs. 49.9), they diverged markedly by T2 (58.2 vs. 35.0) and exhibited dramatically different BCR outcomes. This contrast suggests that the dynamic trend of PNI, rather than the baseline level, is strongly associated with BCR risk.

**Table 2:** Comparison of baseline characteristics among trajectory subgroups.

Characteristic	Overall (*n* = 364)	PHigh (*n* = 169)	PLow (*n* = 146)	Declining (*n* = 49)	*p* value
Age (years)	69.0 (65.0–74.0)	69.0 (64.0–72.0)	70.0 (66.2–75.0)	69.0 (65.0–72.0)	0.009
BMI	23.8 (22.1–25.5)	23.7 (22.1–25.6)	24.0 (22.0–25.6)	23.8 (21.7–24.7)	0.779
Preoperative PSA (ng/mL)	15.4 (10.3–25.6)	14.2 (10.0–23.0)	16.0 (10.4–29.4)	17.9 (10.7–24.1)	0.43
PNI_base	49.5 (46.9–52.1)	51.6 (49.7–53.6)	46.8 (44.9–48.7)	49.9 (48.7–52.0)	<0.001
PNI_T0	49.5 (46.9–52.1)	51.6 (49.7–53.6)	46.8 (44.9–48.7)	49.9 (48.7–52.0)	<0.001
PNI_T1	48.7 (46.3–51.3)	51.0 (48.9–53.1)	46.7 (44.7–48.6)	46.8 (45.5–49.0)	<0.001
PNI_T2	51.0 (45.2–57.8)	58.2 (56.2–60.6)	46.9 (44.7–48.8)	35.0 (33.3–37.0)	<0.001
ISUP grade GG4-5	160 (44.0)	50 (29.6)	88 (60.3)	22 (44.9)	<0.001
Positive surgical margin	90 (24.7)	37 (21.9)	33 (22.6)	20 (40.8)	0.019
Seminal vesicle invasion	51 (14.0)	25 (14.8)	21 (14.4)	5 (10.2)	0.708
Lymph node invasion (LNI)	20 (5.5)	6 (3.6)	11 (7.5)	3 (6.1)	0.295
Lymphovascular invasion (LVI)	68 (18.7)	27 (16.0)	32 (21.9)	9 (18.4)	0.402
Neoadjuvant ADT	51 (14.0)	22 (13.0)	19 (13.0)	10 (20.4)	0.382
Adjuvant therapy	49 (13.5)	20 (11.8)	22 (15.1)	7 (14.3)	0.692
BCR rate	98 (26.9)	18 (10.7)	49 (33.6)	31 (63.3)	<0.001

Continuous variables are presented as median (interquartile range); categorical variables as *n* (%). PNI_T0, PNI_T1, PNI_T2 represent PNI measurements at baseline, 3 months, and 12 months postoperatively.

### Association of trajectory classes with BCR risk

The Kaplan–Meier survival curves showed significant differences in BCR-free survival among the three groups (log-rank *p* = 8.0 × 10^−13^) (Figure 1C): the Declining group had the earliest and lowest survival, the PLow group was intermediate, and the PHigh group had the highest survival. Pairwise comparisons were all significant (all *p* < 0.001). Cox regression results are presented in Table 3. Using PHigh as reference, in the unadjusted model, PLow (HR = 3.62, 95% CI: 2.11–6.23) and Declining (HR = 7.37, 95% CI: 4.11–13.22) both showed significantly increased BCR risk (both *p* < 0.001). After full adjustment, PLow (HR = 2.95, 95% CI: 1.67–5.22, *p* < 0.001) and Declining (HR = 5.80, 95% CI: 3.16–10.64, *p* < 0.001) remained independent risk factors. The proportional hazards assumption was satisfied for the trajectory class Cox model (global *p* = 0.260). The landmark analysis results are summarized in the Sensitivity Analyses section below.

**Table 3:** Cox regression analysis of PNI trajectory classes and BCR risk.

Characteristic	Unadjusted		*p* value	Adjusted		*p* value
	HR	95% CI		HR	95% CI	
PLow vs. PHigh	3.62	2.11–6.23	<0.001	2.95	1.67–5.22	<0.001
Declining vs. PHigh	7.37	4.11–13.22	<0.001	5.8	3.16–10.64	<0.001
PLow vs. Declining	0.49	0.31–0.77	0.002	0.51	0.31–0.83	0.007
(Continuous)	0.94	0.89–0.98	0.008	0.97	0.92–1.03	0.32
Low vs. High PNI	1.66	1.13–2.46	0.01	1.23	0.80–1.90	0.347

HR, hazard ratio; CI, confidence interval. Trajectory class comparisons use PHigh as reference (PLow vs Declining uses Declining as reference). PNI_base (continuous) and Low PNI vs High PNI analyses are based on the full cohort (*n* = 436). Adjusted variables include age, log PSA, pathological T stage, ISUP grade, surgical margin, seminal vesicle invasion (SVI), lymph node invasion (LNI), and lymphovascular invasion (LVI).

### Discrimination and clinical utility of the prediction model

A Cox proportional hazards prediction model incorporating prespecified variables—PNI trajectory classification, log-transformed PSA, surgical margin status, and lymph node metastasis—was applied to the trajectory cohort. The estimated regression coefficients were incorporated into the model as the linear predictor: 1.04 × PLow + 1.57 × Declining + 0.81 × log(PSA) + 1.02 × positive_surgical_margin + 0.84 × lymph_node_metastasis. The ROC curve analysis (Figure 1D) showed that the model had strong discriminative ability, with time-dependent AUCs of 0.823 (95% CI, 0.762–0.883) at 3 years and 0.790 (95% CI, 0.722–0.858) at 5 years; the optimism-corrected 3-year AUC was 0.815 (mean optimism = 0.008) and the optimism-corrected 5-year AUC was 0.781. The IPCW Brier score (Figure 2B) was 0.117 at 3 years (25.8% improvement over the null model, Brier = 0.158) and 0.166 at 5 years. Calibration at 3 years (Figure 1E) yielded slopes of 1.057 (apparent) and 0.989 (optimism-corrected; ideal = 1.0), with intercepts of −0.062 (apparent) and −0.013 (corrected; ideal = 0). At 5 years, the calibration slopes (Figure 1F) were 1.001 (apparent) and 0.940 (corrected), with intercepts of 0.005 (apparent) and 0.041 (corrected), indicating good calibration at both time points. A nomogram for the 3-year BCR-free probability based on the Cox model is provided in Supplementary Figure S1. Internal validation using bootstrap optimism correction (1,000 resamples for calibration, 500 for AUC optimism) confirmed favorable discrimination and calibration. Decision curve analysis showed that the optimal model provided a superior net clinical benefit over treat-all or treat-none strategies across threshold probabilities of 10–40%, suggesting good clinical utility. The comprehensive performance metrics are summarized in Table 4.

**Table 4:** Cox prediction model: performance metrics.

Time horizon	AUC (apparent)	AUC (corrected)	Brier (IPCW)	Improvement over null	Cal slope (app/cor)	Cal intercept (app/cor)
3-year	0.823 (0.762–0.883)	0.815	0.117	25.8%	1.057/0.989	−0.062 / −0.013
5-year	0.790 (0.722–0.858)	0.781	0.166	25.4%	1.001/0.940	0.005/0.041

AUC, area under the time-dependent ROC curve; Brier, IPCW Brier score; Cal, calibration; app, apparent; cor, optimism-corrected (bootstrap *B* = 500 for AUC, *B* = 1,000 for calibration). Improvement over null = percentage reduction in Brier score relative to the null model. Proportional hazards assumption satisfied for the full prediction model (global *p* = 0.69).

### Sensitivity analyses confirming robustness

To verify the robustness of our results, we performed four sensitivity analyses. (1) After excluding patients who received neoadjuvant ADT (*n* = 377, BCR = 104), the trajectory class effects remained robust: PLow adjusted HR = 2.29 (95% CI: 1.26–4.14, *p* = 0.006), Declining HR = 4.73 (95% CI, 2.52–8.87, *p* < 0.001); baseline PNI as a continuous variable remained non-significant (HR = 0.99, *p* = 0.644). (2) After excluding patients who received adjuvant therapy (*n* = 379, BCR = 102), the trajectory effects were even more pronounced: PLow HR = 2.92 (95% CI, 1.56–5.47, *p* < 0.001), Declining HR = 6.38 (95% CI, 3.29–12.39, *p* < 0.001). (3) In the complete-case analysis (*n* = 364, BCR = 98), baseline PNI continuous effect was still not significant (HR = 0.95, *p* = 0.105), whereas trajectory class effects were consistent with the main analysis (PLow HR = 2.95, *p* < 0.001; Declining HR = 5.80, *p* < 0.001). (4) The 12-month landmark analysis (*n* = 340, 80 post-landmark events) confirmed the trajectory-BCR association (Figure 2C): PLow (HR = 2.72, 95% CI: 1.45–5.10, *p* = 0.002) and Declining (HR = 4.79, 95% CI: 2.54–9.02, *p* = 1.24 × 10^−6^) remained independent risk factors, closely consistent with the original adjusted HRs. The time-dependent Cox model showed directionally consistent hazard ranking (PHigh < PLow < Declining). In summary, the association between PNI trajectory class and BCR remained robust across all sensitivity analyses, while the independent predictive value of baseline PNI remained non-significant after multivariable adjustment, further supporting the superior prognostic value of dynamic PNI trajectories over a single baseline measurement.

## Discussion

In this longitudinal trajectory clustering study of high-risk localized prostate cancer, we identified three distinct postoperative PNI trajectories and compared their prognostic value versus static baseline PNI. We found that baseline PNI failed to independently predict BCR after multivariable adjustment (continuous HR = 0.97, *p* = 0.320; dichotomized HR = 1.23, *p* = 0.347), whereas trajectory classes demonstrated strong, graded associations with BCR risk—adjusted HRs for the persistently low and declining groups were 2.95 and 5.80, respectively (both *p* < 0.001). The multivariable Cox model incorporating trajectory information achieved excellent performance (3-year AUC = 0.823, optimism-corrected 0.815; 5-year AUC = 0.790), with good calibration (corrected slopes 0.989 and 0.940 at 3 and 5 years) and Brier scores of 0.117 (25.8% improvement over null) and 0.166. Collectively, these data indicate that longitudinal PNI trend captures host-tumor dynamics more effectively than a single time-point value. The inconsistent findings in prior studies on baseline PNI may partly reflect the loss of dynamic information inherent to single perioperative measurements.

The three trajectory classes identified in this study have distinct clinical and biological implications. The persistently high PNI group (46.4%) maintained a high PNI level throughout (T0 51.6 to T2 58.2) with a BCR rate of only 10.7%, reflecting good preoperative nutritional-immune reserve and postoperative recovery capacity; their tumor biology may be relatively indolent or effectively controlled by host immune surveillance (25). The persistently low PNI group (40.1%) maintained low PNI levels around 46–47 at all three time points, with a BCR rate of 33.6%. This group had older age, higher preoperative PSA, and a very high proportion (72.3%) of high ISUP grade, suggesting that these patients may have been in a state of chronic nutritional depletion and immunosuppression even before surgery, with more aggressive tumors that are difficult to reverse postoperatively. Their risk mechanism may be more related to cumulative damage from “chronic low-level exposure.” The declining PNI group (13.5%) showed a completely different pattern – PNI at T0 was close to the high group (49.9), but dropped sharply to 35.0 at T2, a decrease of about 30%, with a BCR rate as high as 63.3%. This group had a significantly higher rate of positive surgical margins (40.8%), suggesting a potentially higher residual tumor burden. Residual tumor cells may secrete pro-inflammatory cytokines (e.g., IL-6, TNF-α) to activate systemic inflammatory responses, and induce changes in hepatic acute-phase protein synthesis, leading to sustained albumin decline. Meanwhile, expansion of myeloid-derived suppressor cells and regulatory T cells in the inflammatory microenvironment may further suppress peripheral blood lymphocyte proliferation, forming a vicious cycle of “inflammation-malnutrition-immunosuppression” (26–30). These mechanistic speculations are based on existing literature, but we did not directly measure the relevant inflammatory and immune markers in this study; the exact pathways need to be verified by future experimental studies.

We used a two-stage longitudinal trajectory clustering approach to analyze longitudinal PNI data, which offers significant methodological advantages over traditional methods. Traditional time-dependent covariate Cox models assume that all individuals follow the same average trend in PNI over time, ignoring population heterogeneity. In contrast, our two-stage strategy first extracts each patient’s intercept and slope via individual linear regression, and then uses GMM clustering to identify latent subclasses with different trajectory characteristics, without prespecifying a single linear or nonlinear change pattern, which better reflects clinical reality (16, 31). In our study, the 3-class model had an entropy of 1.000, AvePP >0.999, and OCC far above 5, confirming the high reliability and robustness of the trajectory classification. From a clinical perspective, the multivariable Cox model incorporating PNI trajectory class showed robust predictive value, with a 3-year time-dependent AUC of 0.823 (optimism-corrected 0.815) and a 5-year AUC of 0.790. This performance highlights the substantial contribution of dynamic PNI trajectory to BCR risk stratification beyond conventional clinicopathological factors. Decision curve analysis further demonstrated that within the clinically reasonable threshold probability range of 10–40%, the integrated trajectory model provided greater net benefit than the treat-all/none strategies. For clinical translation, future work could develop simplified classification rules based on early postoperative PNI change slopes (e.g., T0 to T1), or use machine learning algorithms for real-time dynamic risk alerts, thereby turning our methodological findings into actionable clinical decision support tools (24).

Previous studies on PNI and prostate cancer prognosis have yielded inconsistent results (13, 32–35). Some reported that low preoperative PNI was an independent risk factor for BCR, while others found negative results. This heterogeneity may stem from differences in the PNI cut-off values used (ranging 44.5–49.2), varying follow-up durations, and sample sizes (32–36). Our study not only confirmed that these controversies may partly arise from the inherent information loss of static measurements, but also demonstrated the robustness of our results through multiple sensitivity analyses. First, regardless of excluding patients who received neoadjuvant ADT or those who received adjuvant therapy, the effect sizes of trajectory classes remained highly significant, ruling out confounding from perioperative and postoperative treatments on PNI measurements and BCR outcomes. The complete-case analysis results were fully consistent with the main analysis, further excluding bias from missing data. Together, these sensitivity analyses form a strong evidence chain supporting that the association between PNI trajectories and BCR is a genuine biological association rather than confounding or chance.

Several limitations should be acknowledged. First, PNI is an arithmetic combination of albumin and lymphocyte count; although easy to obtain, it cannot distinguish between malnutrition-driven and inflammation-driven hypoalbuminemia, nor can it differentiate total lymphocyte count from functional subsets. Future studies incorporating systemic inflammatory markers and immune cell profiling could better dissect the underlying immunobiological mechanisms (37, 38). Second, this was a single-center retrospective cohort with a median follow-up of 38.2 months; although the sample size and event counts were adequate for multivariable analyses and trajectory modeling, generalizability is limited, and longer follow-up is needed to assess associations with distant metastasis and cancer-specific survival (4, 39). Third, our trajectory classification was based on only three measurement time points (baseline, 3 months, and 12 months), capturing the baseline level and overall direction/magnitude of change rather than a continuously measured longitudinal pattern; additional time points could reveal more complex trajectory structures. The term “trajectory” is used as a convenient label for these data-driven change-pattern subgroups. Fourth, the 4-class model was statistically optimal by information criteria (BIC/AIC/aBIC) and BLRT (*p* < 0.0001), but was not selected for the primary analysis because the additional class (*n* = 28, 7.7%) merely subdivided the PHigh group with virtually identical BCR rates (10.7% vs. 10.6%), providing no incremental prognostic information (LRT *p* = 0.996), and the smallest class had insufficient events (~3) for reliable estimation. In a larger cohort, this 4th class might represent a clinically meaningful subgroup. Fifth, the original analysis starting survival from surgery while using 12-month trajectory data could introduce guarantee-time bias; however, our 12-month landmark analysis yielded HRs closely consistent with the original (PLow: 2.72 vs. 2.95; Declining: 4.79 vs. 5.80), confirming that this bias did not materially affect our conclusions. Sixth, this is an observational study; no causal inference analysis was performed, and residual confounding cannot be excluded. The strong association between PNI trajectory and BCR risk does not imply causation.

## Conclusion

Through longitudinal tracking of PNI at three postoperative time points and trajectory clustering analysis in 436 patients with high-risk localized prostate cancer, this study revealed three distinct postoperative PNI change-pattern subgroups. A single baseline PNI could not independently predict BCR after multivariable adjustment (HR = 0.97, *p* = 0.320), whereas PNI trajectory class was strongly and independently associated with BCR risk, with adjusted HRs of 2.95 and 5.80 for the persistently low and declining groups, respectively (both *p* < 0.001). A 12-month landmark analysis confirmed these findings (PLow HR = 2.72, Declining HR = 4.79). The Cox prediction model integrating PNI trajectory class with clinical factors achieved a 3-year time-dependent AUC of 0.823 (optimism-corrected 0.815) and a 5-year AUC of 0.790, with good calibration and Brier scores at both time horizons. These findings, based on an observational cohort without causal inference, support the potential utility of dynamic PNI monitoring for postoperative risk stratification. Patients with persistently low or sharply declining postoperative PNI should be considered at high risk. We recommend establishing a standardized postoperative PNI monitoring protocol and integrating dynamic trajectory assessment into risk stratification systems, while acknowledging the need for multicenter prospective validation and external calibration of the prediction model.

## Data Availability

The original contributions presented in the study are included in the article/supplementary material, further inquiries can be directed to the corresponding author.
