# Supplementary material for: Prognostic value of postoperative dynamic PNI trajectories for biochemical recurrence in patients with high-risk localized prostate cancer: a longitudinal trajectory clustering study
Source: Front Nutr. 2026 Oct 6;13:1963784. doi: 10.3389/fnut.2026.1963784 (PMC13680248; doi:10.3389/fnut.2026.1963784)
Supplement: Supplementary file 1 [file Table_1.docx]

**Supplementary Table S1. Model Selection Comparison (1-6 Classes)**

| **Classes** | **BIC** | **AIC** | **aBIC** | **Entropy** | **Min class (%)** | **Min AvePP** | **BLRT P (vs k-1)** |
| --- | --- | --- | --- | --- | --- | --- | --- |
| **1** | 2092.9 | 2073.4 | 2077.0 | - | 100.0 | 1.000 | - |
| **2** | 1730.8 | 1687.9 | 1695.9 | 1.000 | 13.5 | 1.000 | <0.0001 |
| **3** | 1223.1 | 1156.9 | 1169.2 | 1.000 | 13.5 | 1.000 | <0.0001 |
| **4** | 1117.0 | 1027.4 | 1044.0 | 0.990 | 7.7 | 0.967 | <0.0001 |
| **5** | 1145.5 | 1032.5 | 1053.5 | 0.909 | 7.7 | 0.793 | 0.500 |
| **6** | 1170.0 | 1033.6 | 1058.9 | 0.846 | 7.7 | 0.714 | 0.403 |

These re-analyzed BIC values exactly reproduce the values reported in our manuscript (2-class: 1730.8, 3-class: 1223.1, 4-class: 1117.0 under the standard BIC convention where lower is better), confirming the consistency of our trajectory classification. BIC decreases monotonically from 1-class to 4-class, then increases for 5- and 6-class models, with a clear elbow at 4 classes
