# Supplementary material for: Prognostic value of postoperative dynamic PNI trajectories for biochemical recurrence in patients with high-risk localized prostate cancer: a longitudinal trajectory clustering study
Source: Front Nutr. 2026 Oct 6;13:1963784. doi: 10.3389/fnut.2026.1963784 (PMC13680248; doi:10.3389/fnut.2026.1963784)
Supplement: Supplementary file 2 [file Table_2.docx]

**Supplementary Table S2. Subgroup analysis of baseline PNI and BCR risk**

| **Subgroup** | **Level** | **HR (95% CI)** | **P value** | **Interaction P** |
| --- | --- | --- | --- | --- |
| Age | <65 | 0.88 (0.75–1.05) | 0.161 | 0.384 |
|  | >=65 | 0.98 (0.92–1.03) | 0.430 |  |
| BMI | <24 | 0.99 (0.91–1.07) | 0.716 | 0.539 |
|  | >=24 | 0.96 (0.88–1.04) | 0.290 |  |
| Preoperative PSA | <10 | 1.12 (0.88–1.44) | 0.359 | 0.761 |
|  | >=10 | 0.97 (0.91–1.03) | 0.272 |  |
| Clinical T stage | T1 | 0.78 (0.56–1.07) | 0.123 | 0.947 |
|  | T2 | 0.95 (0.87–1.04) | 0.285 |  |
|  | T3 | 0.99 (0.92–1.08) | 0.872 |  |
| Pathological T stage | T2 | 0.98 (0.9–1.08) | 0.729 | 0.279 |
|  | T3a | 0.92 (0.82–1.02) | 0.134 |  |
|  | T3b | 1 (0.89–1.12) | 0.987 |  |
| ISUP grade | GG1-3 | 0.99 (0.91–1.08) | 0.824 | 0.626 |
|  | GG4-5 | 0.97 (0.9–1.04) | 0.360 |  |
| Surgical margin | Negative | 0.97 (0.9–1.04) | 0.412 | 0.830 |
|  | Positive | 0.98 (0.89–1.08) | 0.665 |  |
| Seminal vesicle invasion | No | 0.98 (0.92–1.04) | 0.530 | 0.619 |
|  | Yes | 0.98 (0.85–1.13) | 0.791 |  |
| Lymph node metastasis | No | 0.95 (0.89–1.01) | 0.110 | 0.212 |
|  | Yes | 1.16 (0.93–1.44) | 0.195 |  |
| Lymphovascular invasion | No | 0.99 (0.93–1.05) | 0.698 | 0.900 |
|  | Yes | 0.95 (0.84–1.08) | 0.422 |  |
| Neoadjuvant ADT | No | 0.99 (0.93–1.05) | 0.644 | 0.667 |
|  | Yes | 0.84 (0.7–1.01) | 0.057 |  |
| Adjuvant therapy | No | 0.98 (0.92–1.04) | 0.433 | 0.633 |
|  | Yes | 1.04 (0.87–1.24) | 0.691 |  |

Note: HR = hazard ratio per 1-unit increase in baseline PNI; CI = confidence interval. Each subgroup was analyzed using multivariable Cox regression adjusting for age, log PSA, pathological T stage, ISUP grade, surgical margin, SVI, LNI, and LVI (excluding the stratifying variable itself). Interaction P tests the null hypothesis of no interaction between PNI_base and the subgroup variable. All interaction P values were >0.05, indicating no significant heterogeneity of the PNI effect across subgroups.
